# Establishing a centralised telehealth service increases telehealth activity at a tertiary hospital

**DOI:** 10.1186/s12913-015-1180-x

**Published:** 2015-12-03

**Authors:** Melinda Martin-Khan, Farhad Fatehi, Marina Kezilas, Karen Lucas, Leonard C. Gray, Anthony C. Smith

**Affiliations:** Centre for Online Health, The University of Queensland, Brisbane, Australia; Centre for Research in Geriatric Medicine, The University of Queensland, Brisbane, Australia; School of Advanced Technologies in Medicine, Tehran University of Medical Sciences, Tehran, Iran

**Keywords:** Telehealth, Telemedicine, Coordination, Hospital

## Abstract

**Background:**

The Princess Alexandra Hospital Telehealth Centre (PAH-TC) is a project jointly funded by the Australian national government and Queensland Health. It seeks to provide a whole-of-hospital telehealth service using videoconferencing and store-and-forward capabilities for a range of specialities. The aim of this study was to investigate whether the introduction of a new telehealth coordination service provided by a tertiary hospital centre increased telehealth activities of a tertiary hospital. Evaluation included service delivery records and stakeholder satisfaction.

**Methods:**

Telehealth service delivery model before and after the establishment of the centre is described as well as the project implementation. The study retrieved data related to the number and scope of previous, and current, telehealth service episodes, to ascertain any change in activity levels following the introduction of the new telehealth coordination service. In addition, using a cross-sectional research design, the satisfaction of patients, clinicians and administrators was surveyed. The survey focused on technical utility and perceived clinical validity.

**Results:**

Introduction of a new centralised telehealth coordination service was associated with an increase in the scope of telehealth from five medical disciplines, in the year before the establishment, to 34 disciplines two years after the establishment. The telehealth consultations also increases from 412 (the year before), to 735 (one year after) and 1642 (two years after) the establishment of the centre. Respondents to the surveys included patients (27), clinicians who provided the consultations (10) and clinical or administrative staff who hosted the telehealth consultations in the remote site (8). There were high levels of agreement in relation to the telehealth option saving time and money, and an important health service delivery model. There was evidence from the remote site that modifying roles to incorporate this new service was challenging.

**Conclusion:**

The introduction of a centralised coordination for telehealth service of a tertiary hospital was associated with the increase in the scope and level of telehealth activity of the hospital. The project and model of health care delivery described in this paper can be adopted by tertiary hospitals to grow their telehealth activities, and potentially reduce costs associated with the delivery of services at a distance.

## Background

Access to healthcare is problematic for people who live at a long distance from a city centre or who are frail and unable to travel (regardless of proximity). In the Australian health system, access to specialist doctors requires a referral from a primary care practitioner (or General Practitioner (GP)). Most patients who are referred to a specialist service- must either take a trip (most often with an escort) to their closest major city, or wait to be seen by a specialist in outreach clinics (very intermittent). The former option is time consuming and costly, and the latter may require the patient to wait for a relatively long time to attend an outreach clinic or it may not be available at all.

Telehealth offers a potential solution for improving access to specialist care because it enables patients and clinicians to interact without the need for travel [1, 2]. Research shows that a high proportion of specialist consultations can be performed remotely via videoconference [3]. However, there are a limited number of studies which have focused on the delivery of a range of health care services to a rural or isolated community [4–6]. Princess Alexandra Hospital (PAH) is a tertiary teaching hospital that serves a population of more than one million residents of the South Brisbane metropolitan area. As part of the role of ‘tertiary teaching hospital’, PAH has an interest and obligation in providing support to clinicians in rural and remote hospitals and their patients. To save the cost and time of a (usually) long trip to major cities for the residents of rural and remote areas, Queensland Health, the funder of public, State owned hospitals, has supplied most public hospitals and health centres with telehealth capabilities in terms of hardware, software, connectivity, and technical support. PAH receives referral patients with tertiary or quaternary care requirements from the entire Queensland state as well as neighbouring states of Australia. Individual departments of the hospital are able to provide telehealth services for their patients through their own offices, which are not specifically designed or appointed for tele-consultation. Despite the availability of the technical infrastructure, the utilisation of telehealth services was generally low.

The Australian Government delivered the Digital Regions Initiative (DRI) during a four year period, from July 2009 to June 2013 to help support access to services in remote locations through the use of advanced communications technologies. It co-funded innovative digital enablement projects with state, territory and local governments through a national partnership agreement. The primary aim of the DRI was to improve the delivery of education, health and/or emergency services in regional, rural and remote Australia. The project described in this paper is one of 14 projects jointly funded through the DRI.

The PAH Telehealth Centre (PAH-TC) was a joint project between the Australian Government, and Queensland health department. It aimed to provide videoconferencing (VC) and store-and-forward capabilities for a range of specialities with clinical staff employed by Queensland health and based at the PAH. The service commenced in July 2012 and included both new telehealth services and the migration of existing PAH telehealth services. The implementation of PAH-TC changed the service delivery model of telehealth in the hospital in terms of coordination and administration. The model of telehealth service delivery before and after implementing the centre is described.

### The model of telehealth service delivery and the establishment of PAH-TC

#### Service delivery model before the establishment of PAH-TC

Before the establishment of the PAH-TC, several hospital departments, including diabetes and endocrinology, cardiology, and orthopaedics were practicing their own independent telehealth clinic in their department to deliver the specialist care or patient education at a distance through the Queensland Health Wide Area Network (WAN). The coordination of telehealth service was administered by the staff of each department. The process of referring patients typically was instigated by General Practitioners who sought specialist opinion on the assessment and management of patients. After being triaged at the department, the patients who were not residing in Brisbane and appeared to be suitable for remote consultation were offered the choice to be seen remotely by a specialist via videoconference. In some cases, the patients who had been visited in outreach clinics were scheduled for follow-up visits via videoconference. Such offers saved the patients the cost and inconvenience of a round trip to the capital city of Queensland or another major city where the required specialist expertise is available. If accepted by the patients, the telehealth appointment was fixed and the patient was directed to the nearest health centre or hospital from where the telehealth session was held. In addition, an arrangement was needed for the staff of the distal clinic to facilitate teleconsultation.

#### PAH-TC project

**Fig. 1:**
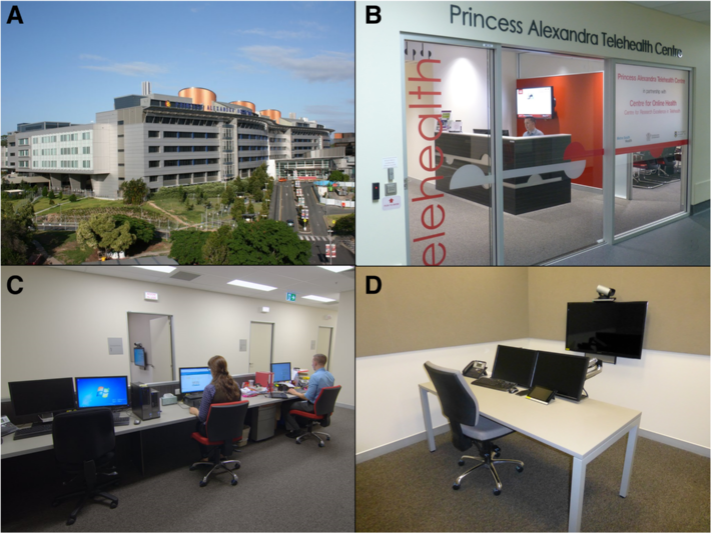
Princess Alexandra Hospital Telehealth Centre - **a** Main building, **b** The Centre entrance, **c** Coordination desk, **d** Telehealth studio

At a cost of around AU$1.6 million, the telehealth centre (PAH-TC) was designed and built at the PAH during eight months and the centre commenced operations in July 2012. The general operating costs for the first two years were AU$800,000. The aim of this project was to establish a centralised telehealth coordination function, focussed on a suite of offices and telehealth studios, but with the intention of supporting telehealth activities across the entire hospital. The project was implemented through a number of key stages: stakeholder consultation; design and construction; installation of telehealth systems and infrastructure; service planning and development of protocols; service coordination; and evaluation. The facility is conveniently situated within the main building of the hospital and contains six custom-designed clinical telehealth consultation rooms; two videoconference-enabled meeting rooms and workspace for staff responsible for telehealth coordination, research and development (Fig. 1). The Centre is equipped with Queensland health department approved videoconferencing systems. The consultation rooms are equipped with Cisco C20 codecs and the meeting rooms with Cisco C40 codecs. Each system has Sony TVs and Cisco InTouch touch panel controllers. The coordination area has a Cisco EX90 tabletop system. All systems are connected to the Queensland Health Private WAN infrastructure. Queensland Health videoconference units communicate with each other using international videoconferencing standards (Table 1).

**Table 1:** Protocols and technical settings used in PAH-TC for videoconferencing

Call standards:	H.323, H.320, SIP
Video protocols:	H.264, H.263, H.261 (minimum video protocol - H.261)
Audio protocols:	G.722, G.722.1, G.723.1, G.728, G.729, AAC-LC, AAC-LD, G.711 (minimum audio protocol - G.711)
Data:	H.239
Encryption:	AES-128
Transmission:	H.225, H.245, H.460

Four high priority clinical disciplines were explicitly targeted within this project: geriatrics; cardiology; dermatology; and endocrinology. Funding was provided for clinical staff to expand their telehealth activities, but the PAH-TC also worked (and continues to work) with all clinical departments to expand their activities. In 2011, the Australian Medicare scheme instigated funding for video-consultation for patients who live in rural communities. This funding explicitly excluded hospital inpatient services. In 2014, Queensland Health introduced new funding for ambulatory and inpatient telehealth events within the public hospital system. To facilitate development, the PAH partnered with the University of Queensland’s Centre for Online Health (COH) to bring to the project extensive experience in telehealth design, operation and evaluation [7]. A formal strategic plan was developed to assist with the “re-engineering” of conventional systems to maximise the use of telehealth for the delivery of specialist clinical services. The remote telehealth locations were established by developing relationships with key stakeholders at each site. A feasibility analysis was carried out to ensure that the required infrastructure and staffing were in place to operate the service. Mobile videoconferencing systems were purchased for the eight distal sites. COH provided additional support to ensure system installations were prioritised; and that appropriate training and support was provided. No construction was required at any distal site except for the installation of wireless infrastructure at two sites.

#### Service delivery model after the establishment of PAH-TC

After the establishment of PAH-TC, several onsite-tours were organised to introduce the centre, its functionalities and capabilities to the physicians and managers of various departments of the hospital as well as external parties. The clinicians who were already practising telehealth were invited to use the facilities available in the centre if preferable. The centre has administrative staff responsible for planning and coordinating telehealth services and streamlining telehealth booking processes. Other clinicians were also encouraged to establish a telehealth practice with the help of administrative and coordination services available. Telehealth clinics could be managed simultaneously in six studios of the centre during business hours, and after hours by appointment. The telehealth centre staff facilitated the VC by ensuring that appointments are scheduled, equipment is operational and connections with remote sites occur seamlessly. In addition, the PAH-TC is utilised to deliver educational programs to a variety of sites across Brisbane and Queensland.

Two main modes of telehealth service delivery are adopted by the centre: 1) Synchronous mode using interactive (real-time) videoconferencing for the patients requiring specialist consultation (e.g. tele-endocrinology clinic); 2) Asynchronous (store-and-forward) mode in which the patients’ data were captured, stored, and transmitted for specialist consultation (e.g. tele-dermatology clinic).

Synchronous mode is mainly used in cardiology, geriatric medicine and endocrinology disciplines. Routine (usually weekly) videoconference clinics are scheduled. A specialist physician remotely consults the patient via videoconference, makes the recommendations to the patients and reports the outcome of the consultation to the referring physician (usually a GP). Expansion plans include the introduction of strategies to help improve attendance rates at telehealth clinics, plus the triaging for telehealth at the point of referral. There is also a plan for the provision and installation of a solution to support the storage and transmission of pre-recorded digital images (e.g. radiology and dermatological) and videos (e.g. ultrasound) from distal sites for reporting at the PAH-TC.

**Fig. 2:**
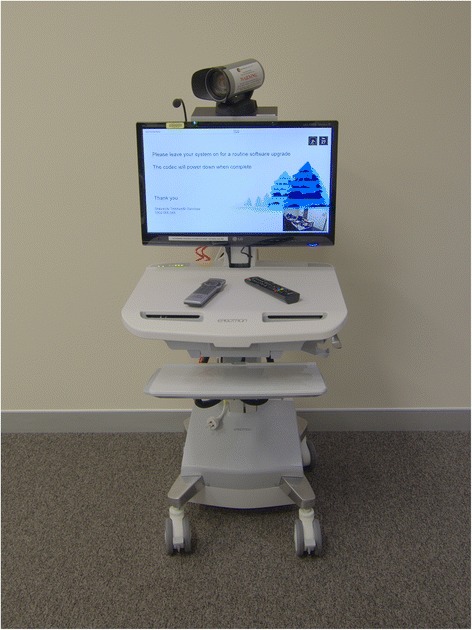
A wireless videoconferencing trolley at a distal site

In the geriatric medicine discipline, routine (weekly) inpatient ward rounds are carried out to small and medium sized rural hospitals. Videoconference links PAH-based specialists to healthcare professionals at the patients’ bedside, deploying mobile wireless VC carts (Fig. 2) for this purpose, followed by multi-disciplinary case conferencing. A geriatrician is recruited to carry out these consultations. Support staff were trained at the distal site to support case preparation and to facilitate implementation of recommendations. Training coincided with the availability of the equipment to ensure that clinicians retained the necessary skills and were familiar with all of the processes associated with the telehealth service (patient assessment systems, referral process, consultation protocols, equipment use and documentation).

Asynchronous mode is mainly used by the tele-dermatology service. A store-and-forward system is used for dermatology requests in which images of the lesions are captured and delivered for specialist consultation. Technical support systems were developed by the PAH Information Technology Services. In addition to the asynchronous mode, routine videoconference clinics are also scheduled in the PAH-TC for dermatology consultations. In these clinics, a dermatologist visits the patients using interactive videoconferencing.

The aim of this study was to identify whether the introduction of a centralised telehealth coordination service increased telehealth activities of a tertiary hospital. The primary research question was whether the establishment of the PAH TC, with its associated capital investment and recurrent funding, resulted in a greater growth in telehealth activity than would have been otherwise expected. The study also explored the extent to which the stakeholders engaged in the various telehealth services are satisfied with the service.

## Methods

An evaluation of the implementation of this service model was carried out to identify if an increase in telehealth activities occurred using two methodologies; comparing the change in service utilisation, and stakeholder feedback.

### Outcome measures

The primary outcome measure of the study was service utilisation. This study retrieved data related to the number and scope of previous telehealth service points for the year before the establishment of the centre, and the first two years of operation of the centre, to ascertain any change in the trend of telehealth activity following the introduction of the centralized coordination by the establishment of the centre. The secondary outcome measure of the study was stakeholder satisfaction by which we aimed to identify the level of satisfaction of the patients, clinicians and administrators with the centrally coordinated telehealth service.

### Data collection

Sources of data included service delivery records, before and after the establishment of the PAH-TC, and stakeholder reported outcomes in relation to the technical and clinical components of the telehealth service. In addition, using a cross-sectional research design, a survey of patients, clinicians and administrators was conducted. The survey focused on technical utility and perceived clinical validity.

### Service utilization

Utilisation data was extracted from the PAH-TC outpatient scheduling system. The following data items were utilised: Data relating to number of telehealth service points, type of service (Video conference or store-and-forward), remote location of service (i.e. the town where the video conference is being received), speciality type (i.e. geriatrics), clinic times (start and finish, reoccurring). The data was extracted in blocks of three months commencing from July 2012 and continuing until 30 June 2014. Utilisation data (from the previous 12 months) prior to the commencement of the PAH-TC was sourced from the hospital administrative systems.

### Stakeholder satisfaction

To establish stakeholder perspectives, all users engaged in a six week period from October 2013 were surveyed. After reviewing the literature three surveys were designed. Each was tailored specifically to the experience of the three stakeholder groups:

- Patient: All adult patients, Any patient under the age of 18 was represented by a parent who responded on their behalf;
- Telehealth clinician: The clinician providing the telehealth service through the PAH-TC;
- Remote clinician and/or administrator: A remote clinician is a staff member involved in facilitating the telehealth service at the remote (distant) site. This may be a nurse, a doctor at a hospital or a general practitioner. It includes general practitioners who referred patients, but were not present at the video conference assessment.

The questions focused on issues relevant to access to specialist support using telehealth: previous use of telehealth; previous requirement to travel for specialist care; willingness to use the telehealth service again and; quality and clarity of the video conference experience. Each question in the survey was scored on a Likert scale of 1-5 (example: Strongly agree; agree; neither agree nor disagree, disagree, strongly disagree). The surveys were pilot tested in a previous study (unpublished), prior to implementation in this study. General demographic data was also collected (gender, age, location, appointment speciality).

Distance from home town to the nearest hospital with VC facilities and distance from home town to the PAH-TC was calculated using driving distance and time from the Whereis website [8], with the payment of relevant tolls included. Individual addresses were not collected so distance was calculated from city centre to city centre. People who resided in the town where the telehealth centre was active were recorded as having travelled zero distance, when in fact there would have been some driving, though it is assumed to be minimal.

#### Statistical analysis

Data was recorded in Excel and analysed in IBM SPSS version 21. Frequency distribution, proportions, range, mean and standard deviation were used for descriptive analysis of data. The results were presented in the form of graphs and tables.

#### Recruitment

De-identified database information was used for the analysis of service utilisation, therefore no individual recruitment was required. To recruit participants for the satisfaction survey, we used the PAH-TC database of patients, telehealth clinicians and remote clinicians/administrators. There were no exclusion criteria, other than a date range for service use. A representative of the PAH-TC contacted each person who utilised the service during the specified study time period (six weeks) and invited them to participate in the survey. Verbal consent was obtained prior to completing the survey over the phone. If preferred, a written survey was provided to the participant for completion (either in person or by mail). Ethics and Governance approval was obtained for the study from the Centres for Health Research and Governance Metro South Hospital and Health Service (HREC/12/QPAH/479; SSA/12/QPAH/481).

## Results

**Fig. 3:**
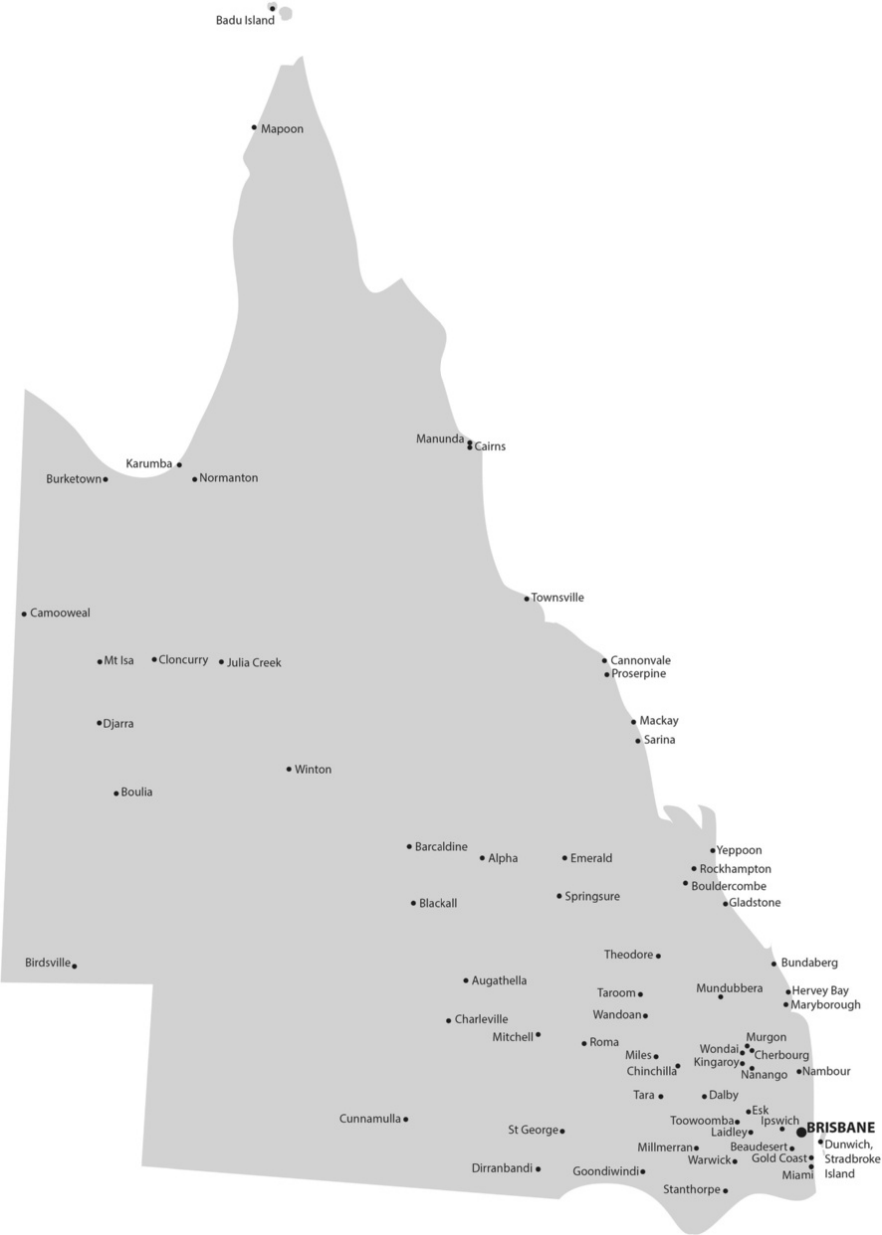
Locality map of the towns receiving telehealth services from the PAH-TC in Queensland, Australia

Prior to the establishment of PAH-TC, telehealth was practiced in five medical disciplines: Cardiology, Endocrinology, Gastro-hepatology, Geriatrics, and Orthopaedics. PAH-TC started operation in July 2012 and during the first year of operation, PAH-TC served video consultations for 18 new disciplines, and total areas of specialized care exceeded 30 medical disciplines and allied health services after two years (Table 2). To date, PAH-TC services have reached 27 regional communities throughout the Queensland state (Fig. 3).

**Table 2:** Medical specialties providing telehealth services at the Princess Alexandra Hospital

	2011–2012 (before implementation)	2012–2013 (year one after implementation)	2013–2014 (year two after implementation)
Medical specialties served by PAH-TC		Dermatology*	Dermatology
		Endocrinology	Endocrinology
		Geriatric	Geriatric
		Haematology*	Haematology
		Orthopaedics	Orthopaedics
			Spinal injury*
Medical specialties that were served both by PAH-TC and outside PAH-TC		Cardiology	Cardiology
		Clinical Pharmacy*	Mental health*
Medical specialties that were served exclusively outside PAH-TC	Cardiology	Community Health Services*	Aboriginal Health Clinic*
	Endocrinology	Ear Nose and Throat Surgery*	Clinical Measurement
	Gastro-Hepatology	Gastroenterology	Clinical Pharmacy
	Geriatrics	General Medicine*	Cognition and Memory*
	Orthopaedics	Genetics*	Community Health Services
		Gynaecology*	Ear Nose and Throat Surgery
		Hydrotherapy*	Gastroenterology
		Infectious Diseases*	General Medicine
		Neurology*	General Surgery*
		Nutrition*	Genetics
		Oncology*	Gynaecology
		Pre-Admission and Pre-Anaesthesia*	Hydrotherapy
		Respiratory*	Infectious Diseases
		Social Work*	Nephrology*
		Speech pathology*	Neurology
			Nutrition
			Occupational Therapy*
			Oncology
			Pain Management*
			Pre-Admission and Pre-Anaesthesia
			Psychogeriatric*
			Rehabilitation*
			Respiratory
			Social Work
			Vascular Surgery*

*New specialty care served via telehealth.

*PAH-TC* Princess Alexandra Hospital - Telehealth Centre

### Service utilisation

**Fig. 4:**
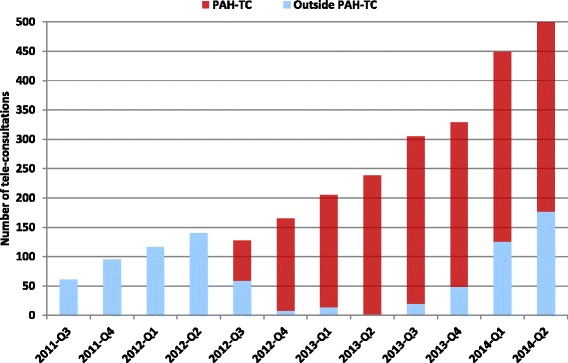
Telehealth activity - one year before and two years after implementation of PAH-TC

One year prior to the establishment of the PAH-TC, 412 telehealth events (occasions of service) were performed throughout the hospital. This figure increased to 735 events one year, and to 1642 events two years after the establishment of the PAH-TC. Overall, continuous growth of telehealth service was seen in the hospital. During the first year after establishment, a high proportion of these events took place in the PAH-TC (ranging from 54 % in the first quarter to 99 % in the fourth quarter), whereas in the second year after establishment, telehealth activities also started to grow in number outside the PAH-TC (from 7 % in the first quarter to 35 % in the fourth quarter). The trend of telehealth activities, within and outside of the PAH-TC is shown in Fig. 4. In addition to video teleconsultation, in which a specialist doctor provided a consultation to the patient via videoconference, there were a number of case discussions undertaken by the specialists in the PAH-TC that are not included in the above figures.

### Stakeholder satisfaction

#### Patients’ access and satisfaction

**Fig. 5:**
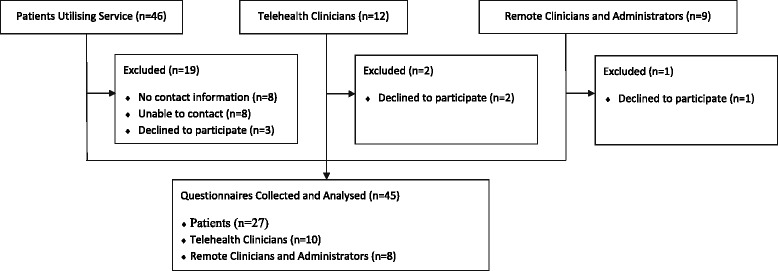
Recruitment and Analysis of Stakeholder Survey (Period 8 October to 8 December 2012)

Forty-six patients were eligible for inclusion in the study (Fig. 5). We managed to contact thirty patients for consent, with three patients declining to participate. Twenty-seven surveys were completed. The majority of the respondents were female (*n* = 16) with an average age of 53 years (Range 16-84, SD 19). Thirteen patients were seen for endocrinology, seven for orthopaedics, four for cardiology and three for geriatrics. The survey was completed by patients residing in 10 different towns, and accessing the telehealth service at six telehealth remote service centres. The majority of patients lived in the same town as the telehealth remote service centre (*n* = 23), the other four lived nearby. For the people living ‘out of town’, the average distance travelled to a telehealth centre was 26 km (Range 25 – 45 km). The average distance between residential town location, and the PA Telehealth Online Centre was 1245 km (Range 150 km – 1820 km).

There was a high level of satisfaction reported by patients in relation to the quality of the videoconference experience and their confidence in the medical advice provided (Table 3; Table 6). Almost 90 % of the patients indicated that without a telehealth service they would have had to travel to a main city centre for the consultation, with 56 % indicating that they had travelled in the past. Almost all patients felt that the telemedicine option had saved them time and money, and that they would utilise the service again if required (Table 5). Important outcomes for the survey included:

- 52 % of people estimated that the need to travel to Brisbane would have cost them in excess of $500 in out of pocket expenses.
- Fifteen patients (56 %) had travelled to a main health centre for a consultation in the past, 13 of those to Brisbane.
- 15 % of patients having to travel to their appointment indicated that their out of pocket expenses were in excess of $500 but most reported a cost averaging between $50 and $300.
- 22 % reported not claiming a refund through the patient travel subsidy scheme, but those who did, they generally received 11–30 % of their total costs back.
- 93 % indicated that telehealth saved them time and money.

**Table 3:** Stakeholder satisfaction (October – December 2012)

Survey Question	Patients	Telehealth Clinician	Remote Clinician
I was satisfied with the quality of the picture (video) during the videoconference	27 (100 %)	10 (100 %)	8 (100 %)
I was satisfied with the quality of the audio (sound) during the videoconference.	27 (100 %)	8 (80 %)	5 (63 %)
I did not experience technical difficulties during the videoconference.*	25 (93 %)	7 (70 %)	2 (25 %)
The videoconference assessment did not make me feel nervous and uncomfortable.*	26 (96 %)	N/A	N/A
I was confident that the clinician could assess my condition via videoconferencing	21 (78 %)	8 (80 %)	N/A
Telehealth improves access to specialist care and improves healthcare delivery	25 (93 %)	10 (100 %)	8 (100 %)
Future Use of Telehealth/Telehealth as a mainstream activity	26 (96 %)	9 (90 %)	8 (100 %)

*Several questions were asked in the negative, but converted to positive responses for analysis.

#### Telehealth clinicians’ satisfaction

Twelve clinicians provided telehealth services during the study period for the PAH Telehealth Centre. Ten completed surveys. The completed surveys represented the following clinical specialities: Endocrinology (*n* = 3); Geriatrics (*n* = 3), Cardiology (*n* = 1); Pharmacy (*n* = 1); Orthopaedics (*n* = 1); and Dermatology (*n* = 1). Five of the consultants were inexperienced (having completed no more than 5 telehealth consults). One consultant had done up to 20 telehealth assessments, and four consultants indicated that had completed more than 20 telehealth consultations.

All doctors felt the quality of the picture was excellent (Table 4 ). The majority of the doctors were happy with the sound quality and had minimal technical issues (70–80 %). These services were all carried out via video conferencing, but one clinician felt that store and forward was a more efficient form of telehealth for dermatology. In general the response was positive with 90 % indicating that telehealth should be a part of mainstream clinical service delivery (Table 6). The most significant problem was case preparation at the remote site and interaction with other clinical staff (hospital doctors or general practitioners) (Table 4). It was felt that the care could be improved if there were stronger protocols in this area.

**Table 4:** Technical – Clinicians and Administrators from the Telehealth Centre (*n* = 10) and the remote sites (*n* = 8)

Question	Clinicians and Administrators	
	Telehealth	Remote
I was able to operate the videoconference equipment with ease	10 (100 %)	8 (100 %)
I found the case preparation material provided by the regional clinician (referring the patient) to be very useful	6 (60 %)	N/A
I found the advice and support provided by the clinicians at the PAH to be very useful	N/A	8 (100 %)
I found the technical and administrative support provided through the PAH Telehealth Centre to be very useful	10 (100 %)	4.3 (75 %)
I could carry out all standard activities required for the assessments during the videoconference	8 (80 %)	N/A

**Table 5:** Patient Economic Survey

Question	Total	Individual Responses
I believe that telehealth enables me to save money and time.	25 (93 %)	
If the telehealth service was NOT available I would have to travel to Brisbane for the consultation.	24 (89 %)	
If the telehealth service was NOT available I or my carer would have to lose..................days off work.	17 (63 %)	
* More than 3 days off work*		*7 (26 %)*
* 3 days or less off work*		*10 (37 %)*
* Not Working/Not Applicable*		*10 (37 %)*
If the video consultation was NOT available and you needed to travel for the consultation, how much money do you think you have had to spend in out-of-pocket expenses for the trip to Brisbane (i.e. fares, meals, accommodation, fuel, childcare arrangements)	26 (96 %)	
* Up to $50*		*3 (11 %)*
* $51 – $300*		*6 (22 %)*
* $301 – $500*		*3 (11 %)*
* More than $500*		*14 (52 %)*
Have you ever needed to travel to a main centre for a consultation in the past?	15 (56 %)	
* Brisbane*		*13 (48 %)*
* Toowoomba*		*1 (4 %)*
* Townsville*		*1 (4 %)*
How much money did the last trip cost your family (for fares, meals, accommodation, fuel, childcare arrangements and other extra family expenses)?		
* Up to $50*		2 (7.5 %)
* $51 – $300*		6 (22 %)
* $301 – $500*		2 (7.5 %)
* More than $500*		4 (15 %)
* Cannot Recall*		*1 (4 %)*
How much money did you get back from the PTSS (Patient Travel Subsidy Scheme)?		
* No Claim Submitted*		*6 (22 %)*
* Less than 10 %*		*2 (7.5 %)*
* 11 % – 30 %*		*4 (15 %)*
* 31 % – 50 %*		*1 (4 %)*
* Cannot Recall*		*2 (7.5 %)*

**Table 6:** Feedback from stakeholders following the use of telehealth specialist service

**Patients using telehealth (remote) site**
‘Good service especially if you live too far away’
‘For other people, living in more remote areas Telehealth is good but not for me’
‘I would prefer face to face consultation’
‘Very happy with the service and with the hospital staff’
‘The TV was too high on the wall and a bit far away, but the service was ok’
‘None of my teleconsultations occurred on time. I had to wait 30–45 min past the appointment time’
‘It’s great! I would hate to have to travel and pay the extra costs for 10 min consultation’
‘Gives me the opportunity to see a specialist here in Mt Isa that otherwise wouldn’t have’
**Staff at the telehealth (remote) site**
‘I was surprised that elderly patients love it. Also, Indigenous patients prefer it and like it as well’
‘Doctors are funded for this role but nurses weren’t so this interferes with ability to do work’
‘There were problems interfacing with booking software causing time delays and extra work at regional locations. In some cases the administration of the bookings was disjointed’
**Clinicians at the tertiary hospital (local) site**
‘Access to previous investigations and pathology results, plus some technical difficulties was an issue’
‘Minimal core preparation at the other end and not being able to engage with GPs was a problem’
‘Having a skilled clinician for assessment at the other end of the line would be most beneficial and improve outcomes’

#### Remote Clinician and Administrator Access and Satisfaction

During the two years of the operation of the PAH-TC, more than 70 towns received at least one (but often a regular) telehealth service (Fig. 3). Remote clinicians and administrators included any staff who assisted with the video conference experience. In this service there were nine remote clinicians or administrators involved. Eight agreed to participate in this survey, from five sites. The staff included two cardiology pharmacists, one endocrinology nurse educator, four geriatric nurse specialists and one telehealth coordinator. Of the eight staff involved, six had previously participated in less than five consults each, one had participated in almost 20, and the final person had participated in more than 20 consultations.

From a technical perspective, everyone agreed that picture quality was good but a high proportion of staff experienced technical difficulties and had problems with sound (Table 3). The staff had no difficulties in operating the equipment from the remote end (Table 4). All staff felt that the advice and support from the telehealth clinician was very useful. At this point in the service implementation, the nursing staff felt that their job descriptions had not been modified to take on this extra role so it was time consuming and challenging to fit in the extra responsibilities without reimbursement. This is a significant issue when new technologies or systems are implemented. Despite these challenges all remote staff agreed that telehealth improved access to health services and they were in strong agreement that telehealth should be delivered as part of mainstream clinical care (Table 6).

## Discussion

The aim of this study was to examine the establishment of a telehealth centre in the Princess Alexandra Hospital in Brisbane, Australia, and its impact on telehealth activities of this tertiary hospital. We also sought to identify whether this effort improved access to specialist care for remote and rural areas of the State of Queensland. Previous research has highlighted the importance of central coordination and support service for successful telehealth [9, 10]. It also has been demonstrated that a centralised telehealth service implies a more organised process, better incentives for clinicians and consequently increased telehealth activity [11–13]. Lack of time for preparation and establishment of routine service has been reported as one of the challenges of telehealth [14]. Centralized coordination and administration of telehealth clinics removed this burden from the staff in individual departments of the hospital, therefore made telehealth less challenging for the clinicians with no prior telehealth experience. Research shows the first experience with telehealth changes the attitude of health care providers towards using this modality of health care [15]. The COH, which is one of the leading telehealth research centres in the world according to publication output, were contracted to assist with the design, establishment and operation of PAH-TC. This strategic planning of consulting an academic centre in the establishment of a central telehealth facility in a tertiary hospital appears to be innovative. Furthermore, adequate relationships were developed with stakeholders at remote sites. Considering the characteristics and dynamics of health care organisations has been reported as an important step in telehealth implementation [16]. Therefore substantial efforts were made to identify the specific needs of each remote site and meet those needs in terms of education, technical support, or organisation.

The PAH-TC initially targeted five medical disciplines and within two years expanded to more than thirty disciplines (Table 2). This approach is similar to the development of other successful and sustainable telehealth networks such as Arkansas e-Link. Arkansas state-wide telehealth network, Arkansas e-Link, is a typical telehealth initiative that has evolved from a limited telehealth service initiated specifically for one medical specialty, and then expanded to other medical disciplines and geographical sites [17]. However, in contrast to Arkansas e-Link, the PAH-TC was established by merging the sporadic telehealth services of the PAH into one centrally coordinated and administered unit. This merging strategy has been reported, in larger scale, in the evolution of Ontario telemedicine network that resulted from the merge of three independent regional networks [18].

The total telehealth activity of the hospital increased from 412 video consultations, during one year prior to the establishment of the centre, to 735 in the first year and then to 1582 in the second year. Although near 90 % of telehealth activities during the first year occurred in the PAH-TC, it is very hard to identify the extent to which the establishment of the centre contributed to this increase. During the first year, the policy of the centre was to encourage the hospital clinicians to conduct their telehealth activities in the centre. This was mainly intended to provide the clinicians with confidence to include telehealth in their routine practice. However, in the second year, the PAH-TC staff encouraged the development of services appropriate to clinical need of various departments of the hospital. The centre has influenced, both directly and indirectly, the uptake of telehealth services delivered from outside the centre. As the result of this policy, the percentage of hospital telehealth activities outside of the centre increased from 7 % in the first quarter of the second year to 24 % in the fourth quarter (Fig. 4).

Video consultation has been increasingly researched in many countries [19]. Satisfaction of stakeholders has been shown crucial for the acceptance and uptake of this modality. We identified three key stakeholders: patients, clinicians, and coordinators/administrators. Stakeholder satisfaction is crucial for service continuity. Our survey showed that satisfaction with the service was generally high among all stakeholders. The findings of this survey confirm the results of previous reports on both providers’ and patients’ satisfaction with telehealth and more specifically with videoconferencing [20, 21]. The findings also demonstrated that patients had savings in terms of time and money by using telehealth.

This study had some limitations. The integrity of the hospital administrative data prior to the implementation of the PAH Telehealth service cannot be confirmed, in that it is possible that telehealth consultations occurred but were not recorded in the system. However, there is a clear continuing upward trend beyond the establishment of reliable data capture systems. Where additional information was known, it was used to supplement the hospital administrative data to increase reliability. It was not possible to identify whether patients would have travelled to PAH for an in-person appointment if the telehealth appointment were not available, so it is not clear if the increased activity resulted in correspondingly improved access to specialist care but anecdotally we are aware that telehealth does not replace in-person visits 1:1, telehealth tends to result in additional appointments for patients who would not have travelled. We were unable to find any validated questionnaire for assessing the satisfaction with video teleconsultation. Therefore we developed the questionnaire based on the review of the literature, utilised in a previous study. There is a need for development of a standard questionnaire for evaluating satisfaction of various stakeholders with video consultation [22].

The observed trends may, of course, be attributable to an overall trend towards greater telehealth activity in Australia, incentivised by national and state government policy and funding adjustments [23]. At this time, we do not have at our disposal comparable data from other hospitals.

The study did not examine clinical effectiveness, cost effectiveness or value for money. There was generous funding available to support this initiative in the first two years of operation, in advance of recent funding changes now offered to other hospitals from Queensland Health.

Overall, it is difficult to be sure that the observed trends were attributable to the PAH-TC or to the operational model of coordination associated with it. However, since there is such limited data available around service models, we considered description of the model and its initial impact to be valuable information to inform further telehealth development.

## Conclusions

Centralisation of the telehealth service in a tertiary hospital can increase the level of activity that is otherwise expected. It also can serves as an incubator for the departments and clinicians with little telehealth experience. The project and model of health care delivery described in this paper can be adopted by tertiary hospitals to grow the scope and level of their telehealth activities and therefore improve access to specialized care. Despite this, the telehealth model does not solve the problem of the support that is required by the local provider to run the VC service.

## Abbreviations

COH: Centre for Online Health (The University of Queensland, Brisbane, Australia)
PAH: Princess Alexandra Hospital (Brisbane, Australia)
PAH-TC: Princess Alexandra Hospital Telehealth Centre
VC: Video Conferencing
